# Polyphenolic Profile, Antioxidant and Anti-Inflammatory Activity of Eastern Teaberry (*Gaultheria procumbens* L.) Leaf Extracts

**DOI:** 10.3390/molecules191220498

**Published:** 2014-12-08

**Authors:** Piotr Michel, Anna Dobrowolska, Agnieszka Kicel, Aleksandra Owczarek, Agnieszka Bazylko, Sebastian Granica, Jakub P. Piwowarski, Monika A. Olszewska

**Affiliations:** 1 Department of Pharmacognosy, Faculty of Pharmacy, Medical University of Lodz, 1 Muszyńskiego St., Lodz 90-151, Poland; E-Mails: piotr.michel@umed.lodz.pl (P.M.); a.m.dobrowolska@wp.pl (A.D.); agnieszka.kicel@umed.lodz.pl (A.K.); aleksandra.owczarek@umed.lodz.pl (A.O.); 2 Department of Pharmacognosy and Molecular Basis of Phytotherapy, Faculty of Pharmacy, Warsaw Medical University, 1 Banacha St., Warsaw 02-097, Poland; E-Mails: agnieszka.bazylko@wum.edu.pl (A.B.); sgranica@wum.edu.pl (S.G.); jakub.piwowarski@wum.edu.pl (J.P.P.)

**Keywords:** *Gaultheria procumbens* L., antioxidant activity, anti-inflammatory activity, UHPLC-MS, HPLC-PDA, phenolic content, phenolic profile, proanthocyanidins, chlorogenic acids, flavonoids

## Abstract

Dry leaf extracts of eastern teaberry (*Gaultheria procumbens* L.) were evaluated as a source of bioactive phytocompounds through systematic activity testing and phytochemical profiling. The antioxidant efficiency was tested using five complementary *in vitro* models (DPPH; FRAP; linoleic acid (LA) peroxidation assay; O_2_^•−^ and H_2_O_2_ scavenging tests) in parallel with standard antioxidants. The 75% methanol extract and its diethyl ether, ethyl acetate (EAF), *n*-butanol and water fractions exhibited the dose-dependent responses in all assays, with the highest capacities found for EAF (DPPH EC_50_ = 2.9 μg/mL; FRAP = 12.8 mmol Fe^2+^/g; IC_50_ for LA-peroxidation = 123.9 μg/mL; O_2_^•−^ SC_50_ = 3.9 μg/mL; H_2_O_2_ SC_50_ = 7.2 μg/mL). The EAF had also the highest anti-inflammatory activity in the inhibition tests of lipoxygenase and hyaluronidase (60.14% and 21.83% effects, respectively, at the concentration of 100 μg/mL). Activity parameters of the extracts correlated strongly with the levels of total phenolics (72.4–270.7 mg GAE/g), procyanidins, and phenolic acids, whereas for flavonoids only moderate effects were observed. Comprehensive UHPLC-PDA-ESI-MS*^3^* and HPLC-PDA studies led to the identification of 35 polyphenols with a procyanidin A-type trimer, quercetin 3-*O*-glucuronide, isomers of caffeoylquinic acids, and (‒)-epicatechin being the dominant components. Significant activity levels, high phenolic contents and high extraction yields (39.4%–42.5% DW for defatted and crude methanol extracts, respectively) indicate the value of eastern teaberry leaves as bioactive products.

## 1. Introduction

The Ericaceae is a large family of flowering plants with nearly worldwide distribution and many non-toxic, edible and medicinal members. The berry fruits of the Ericaceae are regarded as rich sources of bioactive phenolic compounds and like other polyphenol-rich dietary products, have attracted much attention as health-promoting antioxidant agents, exerting protective effects against the accelerated aging and certain oxidative stress- and age-related chronic diseases, including cardiovascular disease, neuro-degenerative disorders, and some types of cancer [1,2,3,4,5,6]. However, despite the fact that among dietary sources, Ericaceae berries are particularly abundant in phenolic antioxidants, they accumulate significantly lower levels of total phenolics (usually not more than 15 mg/g gallic acid equivalents, GAE, of dry weight, DW) than those observed in the leaves (usually not less than 40 mg/g GAE DW) [7,8]. Because of this effect and due to the seasonal availability of fresh berries, the leaves of some Ericaceae species have recently been strongly recommended as cost-effective, alternative sources of phenolic antioxidants with great potential for the prevention of chronic diseases [9,10,11].

*Gaultheria procumbens* L. (eastern teaberry, checkerberry, or American wintergreen) is an Ericaceae species native to North America and cultivated all through the Northern Hemisphere. It is an aromatic, small shrub with evergreen leaves and red berry-like fruits of wintergreen scent [12]. In traditional medicine, various tissues of *G. procumbens* and other *Gaultheria* species are used to treat inflammatory disorders, especially rheumatoid arthritis, swelling pain, chronic tracheitis, cold, and acute and chronic prostatitis [12]. The analgesic and anti-inflammatory activities of these plants are believed to be influenced mainly by salicylic acid derivatives, especially methyl salicylate (essential oil), acting through several mechanisms including some antioxidant effects [13,14].

Multiple oxidative stress-related effects are involved in an inflammatory process, including direct production of oxygen-centered free radicals and other ROS by stimulated immune cells. ROS in turn can activate pro-inflammatory gene expression or initiate free-radical chain reactions impairing the function of biomolecules and causing cellular injury. It is known that plant phenolics [15,16,17], can decrease the ROS level and limit inflammatory response through several mechanisms, especially via direct reduction of free radicals (scavenging action) and other ROS, inhibition of ROS generating pro-inflammatory enzymes, and chelation of transition metal ions [18,19]. Particularly effective are polyhydroxylated phenolics like flavonoids and proanthocyanidins [18]. Some molecules of this type, such as quercetin glycosides (3-*O*-glucoside, 3-*O*-galactoside, and 3-*O*-arabinoside), (+)-catechin, (‒)-epicatechin, procyanidins A2 and B2, have been identified recently in the leaves of *G. procumbens* [20]. Considering their structures, these compounds could significantly affect the antioxidant and anti-inflammatory capacity of *Gaultheria* extracts. However, although preliminary reports for crude methanol leaf extracts of *G. procumbens* indicate promising *in vitro* antioxidant activity [21], no systematic quantitative study exists regarding polyphenols in this species, and no information is available about their impact on the antioxidant and anti-inflammatory activity of the leaf tissue.

Therefore, the aim of this project was to evaluate the value of *G. procumbens* leaves as a source of antioxidant and anti-inflammatory polyphenols. Antioxidant capacity of lipophilic (chloroform) and polar (75% methanol) leaf extracts and their various solvent fractions was investigated by five *in vitro* tests of complementary mechanisms. Moreover, the anti-inflammatory activity of the extracts was studied by two methods based on the inhibition of pro-inflammatory enzymes. The fractionation process was the starting point for statistically assisted identification of the main active components of the leaf tissue. The polyphenolic profile of the plant was investigated by UV-spectrophotometric, UHPLC-PDA-ESI-MS*^3^* and HPLC-PDA-fingerprint methods in relation to the observed activity of the extracts and a series of natural and synthetic antioxidant standards.

## 2. Results and Discussion

### 2.1. Extraction Yield and Total Phenolic Content of G. procumbens Dry Leaf Extracts

Selection of the extracting solvent is an important part of extract preparation as it affects extract composition and hence its activity. In general, alcohol and alcohol-water mixtures are considered to be the most efficient extractants for low-molecular weight polyphenols characterized by high antioxidant potential and good bioavailability [18,22]. Hydroalcoholic extracts might be also considered good representatives of traditional medicine forms like infusions or tinctures as in general they would contain similar range of constituents [23].

**Figure 1:**
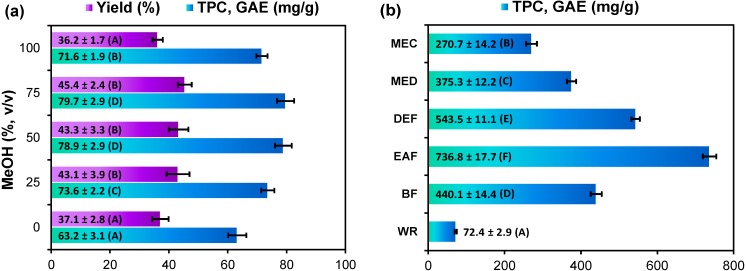
(**a**) Optimisation of the extraction process: yield and recovery of total phenolics from the leaf material dry weight; (**b**) Total phenolic content of dry leaf extracts (TPC).

In this work, the composition of methanol-water mixtures was optimized to maximize recovery and antioxidant activity of the *G. procumbens* leaf extracts. The Folin-Ciocalteu (FC) assay was used to estimate the total phenolic content (TPC) as an indirect measure of antioxidant potential [24]. As presented in Figure 1a, the tested methanol solutions showed statistically different capacities to recover the leaf components. The highest TPC levels were obtained with 75% and 50% methanol, whereas the highest extraction yields (extract weights) were found for 25%, 50% and 75% methanol (45.4% ± 2.4% DW; analytical scale). Since the physical properties of plant extracts and their microbial stability are usually better at elevated alcohol concentration [25], 75% methanol was chosen to prepare the crude leaf extract of *G. procumbens* (MEC). The semi-preparative yield of MEC from 100 g of the dried leaves (42.5%) was in good accordance with the analytical data and similar to the levels (41%–48% DW) reported earlier for the leaves of other Ericaceae species, *i.e.*, *Vaccinium myrtilloides*, *Kalmia angustifolia*, *Arctostaphylos uva-ursi*, *Empetrum nigrum*, and *Arbutus unedo* [9,20,21]. The TPC level in the MEC was 270.7 ± 14.2 mg gallic acid equivalents (GAE)/g DW of the extract (Figure 1b). It is of note, that extraction yields higher than 35% DW are quite rare in nature [21], which confirms the value of Ericaceae leaves as cost-effective sources of phenolic extracts. Furthermore, the highest TPC levels reported earlier for ethanol leaf extracts of Ericaceae plants did not exceed 200 mg GAE/g DW [11] and 290 mg/g DW as expressed as catechin equivalents [9], which puts *G. procumbens* in a very good position comparing to other family members.

Crude leaf extracts usually contain ballast substances (lipoidal or others) that can significantly impair their physical properties, activity and phenolic levels. Due to this fact, there is general tendency in phytotherapy to replace obsolete and ineffective traditional dosage forms with preparations based on standardized dry extracts containing purified and concentrated active constituents [23]. Fractionated extraction is a good way to find solvents that are more specific, for a particular fraction, than water or alcohol and obtain extracts with enhanced activity.

To concentrate the phenolic fraction, the studied leaf material was first defatted with chloroform to obtain the chloroform extract (CHE), and then extracted with the optimized solvent to yield the purified methanol extract (MED). Finally, MED was fractionated between organic solvents to give the fractions of diethyl ether (DEF), ethyl acetate (EAF), *n*-butanol (BF), and the water residue (WR) (for scheme illustrating the extraction process see Figure S1). After removal of the ballast CHE with a significant extraction yield (7.40% DW of the plant material) but low phenolic content (12.7 ± 0.3 mg GAE/g DW), the TPC level in MED increased to 375.3 ± 12.2 mg GAE/g DW with only a slightly lower semi-preparative extraction yield (39.4% DW of the plant material) than observed for MEC. The fractionation led to the further enrichment of polyphenols (Figure 1b) with the highest TPC levels found in EAF (736.8 ± 17.7 mg GAE/g DW) and DEF (543.5 ± 11.1 mg GAE/g DW), which puts the *Gaultheria* extracts among those richest in polyphenols, alongside commercial ethanol extract of grape seed (*ca*. 600 mg GAE/g DW) [26] or the ethyl acetate fractions of green tea and green mate (580 mg/g GAE DW) [27].

All data expressed as mean values ± SD (*n* = 3) represented by error bars. GAE, gallic acid equivalents, calculated per dry weight of the plant material (a) or extract (b), respectively. For each parameter different capital letters indicate significant differences (*p* < 0.05). Extract codes: MEC, crude 75% (*v*/*v*) aqueous methanol extract; MED, purified (defatted) methanol extract; DEF, diethyl ether fraction; EAF, ethyl acetate fraction; BF, *n*-butanol fraction; WR, water residue.

### 2.2. Antioxidant Activity of G. procumbens Dry Leaf Extracts

Oxidative stress is the condition that occurs when the steady-state balance of pro-oxidants to antioxidants is shifted in the direction of the former, creating the potential for organic damage. Pro-oxidants are free radicals, atoms or clusters of atoms with unpaired electrons, among which ROS are prevailing [17,18]. A factor that provides a distinct challenge in the assay of antioxidant capacity is that within biological systems, there are multiple free radical and oxidative molecules, which need to be neutralized by different redox mechanisms. A valuable antioxidant should be thus effective against various pro-oxidants and able to react via both single electron transfer (SET) and hydrogen atom transfer (HAT) basic mechanisms [24].

According to these rules, antioxidant activity of the *Gaultheria* leaf extracts was studied using five complementary *in vitro* assays: the 2,2-diphenyl-1-picrylhydrazyl (DPPH) free radical scavenging and the ferric reducing antioxidant power (FRAP) tests—two the most frequently used SET-type methods, as well as the inhibition of linoleic acid (LA) peroxidation, the superoxide anion (O_2_^•−^) scavenging and the hydrogen peroxide (H_2_O_2_) scavenging tests—three more physiologically-relevant systems which involve the HAT mechanism [21]. Such a study design created the possibility to reflect interactions of the extracts with both nitrogen- and oxygen-centered free radicals (DPPH and O_2_^•−^), non-radical ROS (H_2_O_2_), and transition metal ions (Fe^3+^), as well as to study some of these interactions in the models of lipid oxidation and enzymatic redox systems (xanthine/xanthine oxidase) [19,24].

**Figure 2:**
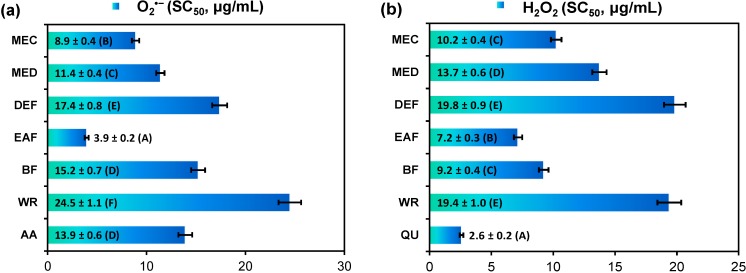
Radical-scavenging activity of *G. procumbens* dry leaf extracts towards: (**a**) superoxide anion (O_2_^•−^) and (**b**) hydrogen peroxide (H_2_O_2_).

In all applied tests, the analyzed extracts displayed a concentration-dependent activity with a wide range of final capacities depending on the extraction solvent (Table 1, Figure 2). The highest activity parameters of the extracts in comparison to phenolic standards were observed in the DPPH and O_2_^•−^ scavenging tests and in the FRAP assay. In all tests EAF was the most active extract. Additionally, in the FRAP and LA-peroxidation assays no statistically significant differences were observed between antioxidant capacities of EAF and DEF (Table 1).

The scavenging activity of EAF against DPPH radical was significantly higher or not statistically different than the activity of several phenolic standards including industrial antioxidants BHA, BHT, and TBHQ (Table 1). In the O_2_^•−^ scavenging test, EAF was more active than ascorbic acid (Figure 2a), and the ability to react directly with O_2_^•−^ was confirmed for the extracts by no inhibitory effect on xanthine oxidase in the simultaneous monitoring of uric acid production. Activity parameters of the extracts in the H_2_O_2_ quenching test (SC_50_ values, Figure 2b) were significantly worse than that of standard quercetin (QU). However, at the concentration of 20 µg/mL the most active EAF exhibited similar scavenging percentage (results not shown). Moreover, when comparing with other plant extracts tested in the same conditions, the title extracts exhibited significantly higher activity against both O_2_^•−^ and H_2_O_2_ [28]. This activity is of great relevance for potential health benefits of eastern teaberry extracts, as both molecules are the most physiologically important ROS implicated in various oxidative stress-related pathologies including inflammatory diseases, ischemia-reperfusion injury, cancer, and the aging process [29].

**Table 1:** Comparison of antioxidant activity of *G. procumbens* dry leaf extracts and standard antioxidants in DPPH, FRAP and inhibition of linoleic acid peroxidation tests *^a^*.

Analyte	Radical Scavenging Activity *^b^*	Reducing Power *^c^*	LA-Peroxidation *^d^*
	DPPH (EC_50_, µg/mL)	FRAP (mmol Fe^2+^/g)	(IC_50_, µg/mL)
**MEC**	8.35 ± 0.28 *^G^*	4.58 ± 0.24 *^B^*	175.98 ± 7.78 *^H^*
**MED**	6.67 ± 0.43 *^F^*	5.97 ± 0.21 *^C^*	207.98 ± 9.47 *^I^*
**DEF**	4.34 ± 0.24 *^E^*	**12.50 ± 0.84** *^F^*	**109.39 ± 5.36** *^G^*
**EAF**	**2.90 ± 0.15** *^C^*	**12.77 ± 0.76** *^F^*	**123.94 ± 6.11** *^G^*
**BF**	4.94 ± 0.25 *^E^*	8.17 ± 0.48 *^D^*	164.77 ± 5.77 *^H^*
**WR**	30.91 ± 1.43 *^H^*	1.46 ± 0.08 *^A^*	651.85 ± 20.21 *^J^*
**CA**	2.17 ± 0.11 *^B^*	25.37 ± 0.44 *^I^*	69.68 ± 0.70 *^F^*
**CHA**	4.42 ± 0.13 *^E^*	18.04 ± 0.79 *^H^*	52.47 ± 2.03 *^E^*
**QU**	1.63 ± 0.07 *^A^*	36.02 ± 1.10 *^J^*	48.51 ± 1.74 *^D^*
**RT**	3.44 ± 0.09 *^D^*	11.89 ± 0.70 *^E,F^*	67.73 ± 0.34 *^F^*
**BHA**	2.90 ± 0.14 *^C^*	16.13 ± 0.83 *^G^*	14.33 ± 0.70 *^A^*
**BHT**	6.54 ± 0.28 *^F^*	18.89 ± 0.42 *^H^*	21.58 ± 0.95 *^B^*
**TBHQ**	2.73 ± 0.12 *^C^*	15.50 ± 0.71 *^G^*	36.53 ± 1.04 *^C^*
**TX**	4.34 ± 0.22 *^E^*	10.83 ± 0.32 *^E^*	22.45 ± 1.10 *^B^*

*^a^* Results are the mean values (±SD for replicates) calculated per dry weight of the extract or standard: CA, (+)-catechin; CHA, 5-*O*-caffeoylquinic acid (chlorogenic acid); QU, quercetin; RT, rutin; BHA, butylated hydroxyanisole; BHT, 2,6-di-*tert*-butyl-4-methylphenol; TBHQ, *tert*-butylhydrochinon; TX, Trolox^®^. For extract codes see Figure 1. Different superscripts (capitals) in each column indicate significant differences in the means at *p* < 0.05. Bolded values indicate the best antioxidants among extracts; *^b^* Radical-scavenging efficiency expressed as EC_50_, effective concentration, amount of antioxidant needed to decrease the initial DPPH concentration by 50% (*n* = 2 × 5 × 1); *^c^* Ferric reducing antioxidant power (*n* = 2 × 5 × 1); *^d^* Ability to inhibit linoleic acid (LA) peroxidation expressed as IC_50_, inhibition concentration, amount of antioxidant needed to decrease the LA-peroxidation by 50%, *n* = 3 × 1.

All data expressed as mean SC_50_ values ± SD (*n* = 3) represented by error bars. For each parameter different capital letters indicate significant differences (*p* < 0.05). Scavenging efficiencies (SC_50_), amounts of antioxidants needed to decrease the initial ROS concentration by 50% (*n* = 3 × 1). Extract codes are given in Figure 1. Positive standards: AA, ascorbic acid; QU, quercetin.

The FRAP activity of the most active extracts was comparable to the activity of rutin (RT) and synthetic vitamin E (Trolox, TX), but in the same time it was 1.5–3 times lower than those of the other standards (Table 1). For small molecular phenolics, such as QU and catechin (CA), their extremely high FRAP activity expressed in weight units might have been partially affected by low molecular mass and better reaction kinetics in comparison to macromolecular constituents [30], especially tannin-type proanthocyanidins abundant in *Gaultheria* extracts (see Section 2.4 and Section 2.5). On the other hand, the slow reaction rate of plant extracts may imply an ability to retain or even increase their reducing power with time, and might thus signify a longer protecting effect against oxidative damage *in vivo* [30].

The chain-breaking activity studies in the LA-peroxidation assay revealed relatively the lowest capacities of the leaf extracts in comparison to other applied tests (Table 1). The most active EAF and DEF showed even up to eight times higher IC_50_ values than lipophilic antioxidants BHA and BHT used as preservatives of industrial lipoidal products. On the other hand, the polar standards, *i.e.*, QU, RT and CA, acted only up to two times stronger than EAF and DEF, indicating that the observed activity differences between analytes may be primarily attributed to their hydrophobicity and affinity restrictions to the oxidized double bonds of LA.

Correlation studies of the antioxidant data matrix including the TPC levels (Table 2) revealed strong linear relationships (|*r*| ≥ 0.7) between most of the tested variables. Some of these trends were statistically significant at α = 0.05, with the most substantial correlations found between activity parameters from the DPPH and LA-inhibition tests, as well as between those from the scavenging tests of O_2_^•−^ and H_2_O_2_. Moreover, in several cases, e.g., for the correlation between the DPPH EC_50_ and FRAP values, strong effects were observed with the probability (*p*) values within the range 0.05–0.10. Although not allowing unambiguous confirmation of direct correspondence between these variables, the results may nevertheless indicate quite universal antioxidant character of the analyzed extracts. As the fractionation process usually results in the separation of phenolics of different structures and activities [31], the strong correlations between the results of the assays varying with regard to their basic reactions are uncommon [24]. The significant relationships found between activity parameters of the *G. procumbens* fractionated extracts in some of the applied test systems, especially in the DPPH, LA-peroxidation and FRAP assays, were thus of note. For further inquiry on this matter, the parallel correlation studies were run for the standards. The linear relationships between activity parameters of the standards in three above mentioned tests were significantly weaker (|*r*| ≤ 0.455), *p* ≥ 0.25) than those observed for *Gaultheria* extracts. The substantially higher |*r*|*-*values for eastern teaberry extracts might be explained by some additive or synergistic effects of their antioxidant constituents and confirm their versatility in different redox reactions. Similar effects, often observed for plant extracts, are usually affected by complementary activity, regeneration mechanisms, and formation of stable intermolecular complexes between individual antioxidants [32].

Linear correlations between antioxidant activity parameters and TPC levels are frequently accepted as convincing evidence for determinative impact of phenolics on the tested capacity [30]. In the case of *Gaultheria* extracts, very strong and statistically significant effects were found in the DPPH and FRAP tests (Table 2). For other assays these trends were less evident (|*r*| ≥ 0.7, *p* > 0.05) or negligible (|*r*| < 0.5, *p* > 0.05), suggesting that some aspects of the tested activity could be partially related to non-phenolic compounds. In this context, it was important that strong DPPH scavenging activity was reported previously [14] for volatile lipophilic fraction (essential oil) of *G. procumbens* leaves (EC_50_ = 30.61 mg/mL *versus* 48.52 mg/mL for gallic acid). On the other hand, considering composition of the oil (96.9% methyl salicylate [14]) and structure of the main component (lack of free hydroxyl groups) these results are controversial. Therefore, in the present study, we decided to verify the DPPH scavenging capacity of volatile and non-volatile lipophilic fractions of the leaves. In parallel tests, the activity of CHE (EC_50_ = 154.2 ± 7.7 µg/mL) and methyl salicylate (no effect up to the concentration of 0.4 g/mL) was very weak or negligible in comparison to gallic acid (0.95 ± 0.05 µg/mL) and the polar extracts (Table 1), which confirmed the determinative impact of non-volatile polar compounds on the antioxidant activity of eastern teaberry leaves.

**Table 2:** Correlation (*r*) coefficients and probability (*p*) values of estimated linear relationships between antioxidant activity parameters and total phenolic contents of *Gaultheria* extracts *^a^*.

*r* (*p*) for:	DPPH EC_50_	FRAP	LA-Inh IC_50_	O_2_^•−^ SC_50_	H_2_O_2_ SC_50_
**DPPH EC_50_**	―	−0.7822 (0.066)	0.9919 (0.000) *	0.7450 (0.089)	0.5579 (0.250)
**FRAP**	−0.7822 (0.066)	―	−0.7744 (0.071)	−0.4957 (0.317)	−0.2574 (0.622)
**LA-Inh IC_50_**	0.9919 (0.000) *	−0.7744 (0.071)	―	0.7297 (0.100)	0.5185 (0.292)
**O_2_^•−^ SC_50_**	0.7450 (0.089)	−0.4957 (0.317)	0.7297 (0.100)	―	0.8203 (0.046) *
**H_2_O_2_ SC_50_**	0.5579 (0.250)	−0.2574 (0.622)	0.5185 (0.292)	0.8203 (0.046) *	―
**TPC (GAE)**	−0.8255 (0.043) *	0.9604 (0.002) *	−0.7970 (0.058)	−0.6800 (0.137)	−0.4751 (0.341)

*^a^* Activity parameters and total phenolic contents (TPC) were defined in Table 1 and Figure 1 and Figure 2. Values marked with an asterisk are statistically significant at α = 0.05.

### 2.3. Anti-Inflammatory Activity of G. procumbens Dry Leaf Extracts

Inflammation is a part of the immune system’s response to harmful stimuli controlled by several cellular enzymes, among which lipoxygenases and hyaluronidases play an important role. Therefore, the inhibition of both enzymes is frequently used as a criterion of anti-inflammatory potential [33].

**Figure 3:**
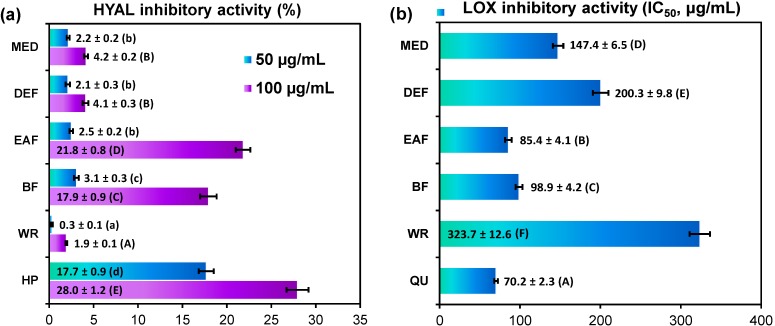
Anti-inflammatory activity of *G. procumbens* dry leaf extracts: inhibitory activity on: (**a**) hyaluronidase and (**b**) lipoxygenase.

In the present study, inhibitory effects on a model hyaluronidase and lipoxygenase were tested *in vitro* with dose-dependent responses observed for all analyzed leaf extracts (Figure 3). The strongest inhibitory potential towards hyaluronidase was demonstrated by EAF and BF with the inhibitory ratio of 21.83% ± 0.82% and 17.93% ± 0.89%, respectively, at the concentration level of 100 µg/mL (Figure 3a). At the same conditions, the inhibitory ratio for positive heparin control was 27.95% ± 1.24%. The other tested extracts showed weak effect on enzyme activity with the inhibitory ability less than 5%.

The extracts were in general more effective inhibitors of lipoxygenase than hyaluronidase, but the activity order was similar in both assays. The highest inhibitory activity towards lipoxygenase was again observed for EAF and BF with the IC_50_ values of 85.41 ± 4.13 µg/mL and 98.88 ± 4.17 µg/mL, respectively, whereas the IC_50_ value for positive quercetin control was 70.16 ± 2.25 µg/mL. The other extracts were less active (Figure 3b). At the concentration level of 100 µg/mL, the inhibitory ratio was in the range of 14.16%–60.14%, depending on the extract.

The measured IC_50_ data for lipoxygenase inhibition (LOX IC_50_) correlated with the DPPH EC_50_ values (*r* = 0.8957, *p* = 0.04), O_2_^•−^ SC_50_ (*r* = 0.8665, *p* = 0.06), H_2_O_2_ SC_50_ scores (*r* = 0.8679, *p* = 0.06), and IC_50_ values for LA-peroxidation test (*r* = 0.7148, *p* = 0.06), indicating that anti-inflammatory and antioxidant activity of *Gaultheria* extracts are closely related. The relatively high correlation between LOX IC_50_ and TPC values (*r* = −0.8175) with low statistical significance (*p* = 0.09) may suggests in turn that both phenolic and non-phenolic compounds are co-responsible for the anti-inflammatory activity of the extracts, but probably with the major role of polyphenols.

All data expressed as mean values ± SD (*n* = 3 × 1) represented by error bars. IC_50_, 50% inhibition of enzyme activity. For each parameter different letters in parentheses indicate significant differences (*p* < 0.05). For extract codification see Figure 1. Positive standards: HP, heparin; QU, quercetin.

### 2.4. Qualitative UHPLC-PDA-ESI-MS*^3^* Profiling of G. procumbens Leaf Phenolics

**Figure 4:**
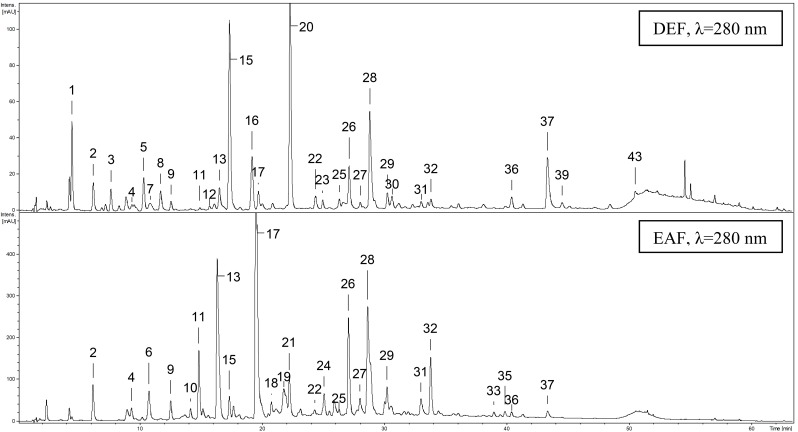
Representative UHPLC-UV chromatograms of diethyl ether (DEF) and ethyl acetate (EAF) extracts. Peak numbers refer to those implemented in Table 3.

The qualitative UHPLC-PDA-ESI-MS*^3^* survey on the leaf extracts revealed the presence of over forty phenolic constituents (UHPLC peaks **1**–**44**), thirty five of which were fully or tentatively identified in contrast to only nine compounds described previously [20]. The main identification data of all detected peaks and the UHPLC fingerprints of the most active extracts are presented in Table 3 and Figure 4, respectively (for full MS*^3^* data see Table S1). According to the spectral profiles, three major groups could be distinguished among analytes including phenolic acids and related quinic acid pseudodepsides, proanthocyanidins, and flavonoids.

**Table 3:** Phenolic analytes detected in *G. procumbens* leaf extracts by UHPLC-PDA-ESI-MS*^3^*.

Peak	Analyte	R_t_ (min)	UV λ_max_ (nm)	[M−H]^−^ (*m*/*z*)	Formula	Extract
**1**	protocatechuic acid (PCA) *^a^*	4.4	295	153	C_7_H_6_O_4_	DEF
**2**	3-*O*-caffeoylquinic acid (NCHA) *^a^*	6.2	325	353	C_16_H_18_O_9_	all
**3**	*p*-hydroxybenzoic acid (*p*HBA) *^a^*	7.6	254	137	C_7_H_6_O_3_	DEF
**4**	3-*O*-*p*-coumaroylquinic acid *^a^*	9.3	310	337	C_16_H_18_O_8_	DEF, EAF, BF
**5**	vanillic acid *^a^*	10.3	260, 291	167	C_8_H_8_O_4_	DEF
**6**	5-*O*-caffeoylquinic acid (CHA) *^a^*	10.7	325	353	C_16_H_18_O_9_	MED, EAF, BF, WR
**7**	(+)-catechin (CA) *^a^*	10.9	280	289	C_15_H_14_O_6_	MED, DEF
**8**	caffeic acid (CFA) *^a^*	11.7	325	179	C_9_H_8_O_4_	DEF
**9**	4-*O*-caffeoylquinic acid (CCHA) *^a^*	12.6	325	353	C_16_H_18_O_9_	all
**10**	3-*O*-feruloylquinic acid	14.2	325	367	C_17_H_20_O_9_	EAF
**11**	procyanidin B-type dimer	14.9	280	577	C_30_H_26_O_12_	MED, DEF, EAF, BF
**12**	unknown compound	15.7	254	481		MED, DEF, BF
**13**	(−)-epicatechin (ECA) *^a^*	16.5	280	289	C_15_H_14_O_6_	MED, DEF, EAF, BF
**14**	4-*O*-*p*-coumaroylquinic acid	16.6	310	337	C_16_H_18_O_8_	BF
**15**	unknown compound	17.3	280	559		all
**16**	*p*-coumaric acid (*p*CA) *^a^*	19.1	310	163	C_9_H_8_O_3_	DEF
**17**	procyanidin A-type trimer (PA)	19.8	280	863	C_45_H_36_O_18_	MED, DEF, EAF, BF
**18**	procyanidin B-type trimer	20.8	280	865	C_45_H_38_O_18_	MED, EAF, BF
**19**	procyanidin A-type dimer	22.1	280	575	C_30_H_24_O_12_	MED, EAF, BF
**20**	unknown compound	22.2	267, 298	639		DEF
**21**	caffeoylquinic acid derivative	22.4	325	391		MED, EAF
**22**	unknown compound	24.3	280	473		DEF, EAF
**23**	quercetin pentoside-glucuronide	24.9	257, 356	609	C_26_H_26_O_17_	MED, DEF, BF, WR
**24**	procyanidin A-type trimer	25.1	280	863	C_45_H_36_O_18_	EAF
**25**	unknown compound	26.3	280	451		DEF, EAF
**26**	quercetin 3-*O*-galactoside ( HY) *^a^*	27.2	254, 353	463	C_21_H_20_O_12_	MED, DEF, EAF, BF
**27**	quercetin 3-*O*-glucoside ( IQ) *^a^*	28.1	256, 353	463	C_21_H_20_O_12_	all
**28**	quercetin 3-*O*-glucuronide ( MQ) *^a^*	28.8	256, 356	477	C_21_H_18_O_13_	all
**29**	quercetin 3-*O*-arabinoside (GV) *^a^*	30.3	258, 356	433	C_20_H_18_O_11_	MED, DEF, EAF, BF
**30**	quercetin derivative	30.6	258, 354	333		DEF
**31**	kaempferol 3-*O*-glucuronide	33.0	265, 349	461	C_21_H_18_O_12_	all
**32**	quercetin 3-*O*-glucuronide methyl ester	33.9	265, 356	491	C_22_H_20_O_13_	MED, DEF, EAF, BF
**33**	kaempferol 3-*O*-glucuronide methyl ester	38.8	265, 348	475	C_22_H_20_O_12_	EAF
**34**	unknown compound	39.0	286, 326	409		BF
**35**	kaempferol 3-*O*-glucoside ( AG) *^a^*	39.9	265, 345	447	C_21_H_20_O_11_	EAF
**36**	unknown compound	40.3	280	451		DEF, EAF
**37**	quercetin (QU) *^a^*	43.3	255, 364	301	C_15_H_10_O_7_	DEF, EAF
**38**	unknown compound	43.7	286, 326	409		BF
**39**	unknown compound	44.4	280	435		DEF
**40**	quercetin 3-*O*-pentoside-glucuronide butyl ester	4.4	295	153	C_30_H_34_O_17_	BF
**41**	quercetin 3-*O*-glucuronide butyl ester	6.2	325	353	C_25_H_26_O_13_	BF
**42**	kaempferol 3-*O*-pentoside-glucuronide butyl ester	7.6	254	137	C_30_H_34_O_16_	BF
**43**	kaempferol (KA) *^a^*	9.3	310	337	C_15_H_10_O_6_	DEF
**44**	kaempferol 3-*O*-glucuronide butyl ester	10.3	260, 291	167	C_25_H_26_O_12_	BF

*^a^* Identified with authentic standards. R_t_, retention times. UV λ_max_, absorbance maxima in PDA spectra. [M−H]^−^, pseudomolecular ions in MS spectra recorded in a negative mode. For systematic names of flavonoid standards see Section 3.1. For extract codes see Figure 1.

#### 2.4.1. Phenolic Acids and Related Phenolic Compounds

The MS/MS profiles of quinic acid monoesters are well studied and the locations of cinnamoyl groups in a pseudodepside structure can be easily determined based on the relative intensities of diagnostic MS*^n^* ions [34]. Among this group, compounds **2**, **6** and **9** (with the same UV maxima at 325 nm) giving parent [M−H]^−^ ions at *m*/*z* 353 were detected in all extracts. The MS*^2^* spectra of **2** and **6** showed fragment ions at *m*/*z* 191 as base peaks and an intense (>40% of base peak intensity) or weak (<5% of base peak) secondary ions at *m*/*z* 179, respectively. Compound **9** exhibited distinctive MS*^2^* behavior with a dehydrated base peak at *m*/*z* 173. Based on hierarchical discrimination key [34] and spiking experiments using standards, both commercial and prepared in our laboratory [35], compounds **2**, **6**, and **9** were characterized as 3-, 5- and 4-*O*-caffeoylquinic acids, respectively. In the case of compound **21**, fragmentation of a pseudomolecular ion at *m*/*z* 391 gave fragment ion at *m*/*z* 179 as a secondary peak in MS*^2^* spectrum, which represents typical caffeic acid-derived ion [34], observed for compounds **2** and **6**. Therefore, compound **21** was identified tentatively as a caffeoylquinic acid derivative.

Three quinic acid derivatives containing *p*-coumaroyl and feruloyl rests in their structures were also found. Compounds **4** and **14** showed [M−H]^−^ ions at *m*/*z* 337 and the MS*^2^* base fragmentation peaks derived from the cinnamic acid moiety at *m*/*z* 163 and *m*/*z* 173, which led to the identification of 3- and 4-*O*-*p*-coumaroylquinic acids, respectively. Compound **10** displayed a [M−H]^−^ ion at *m*/*z* 367 and a MS*^2^* base fragment ion at *m*/*z* 193, which allowed identification of 3-*O*-feruloylquinic acid [34].

Simple derivatives of benzoic (**1**, **3** and **5**) and cinnamic acids (**8** and **16**) were detected in DEF only and identified by direct comparison with reference standards and literature data [36] as protocatechuic, *p*-hydroxybenzoic, vanillic, caffeic and *p*-coumaric acids, respectively.

#### 2.4.2. Flavan-3-ols (Catechins and Proanthocyanidins)

Peaks **7**, **11**, **13**, **17**–**19**, and **24** (with UV maxima at 280 nm) were classified as mono- to oligomeric procyanidins. Compounds **7** and **13** showed [M−H]^−^ ions at *m*/*z* 289 and MS*^2^* ions at *m*/*z* 245 and *m*/*z* 205, respectively. Standard spiking experiments and literature data [37] led to the identification of (+)-catechin (CA) and (‒)-epicatechin (ECA). Compound **11** with a parent ion [M−H]^−^ at *m*/*z* 577, yielding MS*^2^* diagnostic fragments at *m*/*z* 451, 425, 407 and 289, and compound **19** with a [M−H]^−^ signal at *m*/*z* 575 and MS*^2^* fragment ions at *m*/*z* 499, 423 and 289 were identified as procyanidin B- and A-type dimers, respectively. According to [20], these are probably procyanidins B2 and A2. Pseudomolecular ions [M−H]^−^ at *m*/*z* 863 with the relevant MS*^2^* fragmentation indicated the presence of procyanidin A-type trimers (**17**, **24**), whereas a parent [M−H]^−^ ion at *m*/*z* 865 with base daughter MS*^2^* peaks at *m*/*z* 713 and *m*/*z* 577 allowed identification of a procyanidin B-type trimer (**18**) [37]. In the group of proanthocyanidins, 12 individuals were found, with the dominant ECA (**13**) and the A-type procyanidin trimer (**17**).

#### 2.4.3. Flavonoids

Peaks **23**, **26**–**33**, **35**, **37** and **40**–**44** with two UV maxima at approximately 250–267 and 355–365 nm were classified as flavonoids. Compounds **37** and **43** were identified with standards as free aglycones quercetin (QU) and kaempferol (KA), respectively. Glycosides were found by detection of neutral losses typical for sugar moieties (162 for hexoses, 132 for pentoses, and 176 for hexuronic acids). In the case in which one of the neutral loss masses was found, MS*^3^* fragmentation was performed to identify the aglycone moiety [38]. Peaks **26**–**29** with the fragment ion in MS*^2^* spectra at *m*/*z* 301 (typical for QU) were identified with authentic standards as hyperoside (**26**, HY), isoquercitrin (**27**, IQ), miquelianin (**28**, MQ), and guajiverin (**29**). Due to the corresponding MS/MS profile, compound **30** was identified tentatively as a quercetin derivative. Similarly, peaks **31** and **35** with the MS*^2^* fragment ions at *m*/*z* 285 (typical for KA) were classified as kaempferol 3-*O*-glucuronide and astragalin (AG), respectively. In the case of peak **23**, a flavonoid diglycoside, the MS spectra in a positive mode revealed the neutral losses characteristic for a pentose (−132 amu), cleavage of glucuronic acid (−176 amu) and the aglycone signal at *m*/*z* 303. Based on MS*^2^* and MS*^3^* fragmentation patterns and the published data [39], **23** was identified as a quercetin pentoside-glucuronide. Peaks **32**, **41** and **40** gave the parent ions [M−H]^−^ at *m*/*z* 491, 533 and 665, respectively. Their MS spectra in a negative mode revealed the cleavage of glucuronic acid (−176 amu) and the aglycone signal at *m*/*z* 301. Furthermore, the neutral losses distinctive for methyl group (−14 amu), butyl group (−56 amu), and butyl group with pentose (−132 amu) were observed for compounds **32**, **41** and **40**, respectively. On that basis [40], they were identified as quercetin 3-*O*-glucuronide methyl ester, quercetin 3-*O*-glucuronide butyl ester and quercetin 3-*O*-pentoside-glucuronide butyl ester. Compounds **33** ([M−H]^−^ at *m*/*z* 475), **44** ([M−H]^−^ at *m*/*z* 517) and **42** ([M−H]^−^ at *m*/*z* 649) exhibited parallel fragmentation patterns as the latter ones and were recognized as analogous kaempferol derivatives. It is of note that the butyl esters **40**–**42** and **44** were observed exclusively in BF obtained after sequential liquid-liquid extraction of MED. Their absence in the mother extract suggested that they were artifacts formed during extraction. Similar conclusion could be made for methyl esters (compounds **32** and **33**) per analogy.

The study proved the presence of 16 flavonoids, among which compounds **26**–**28**, **31**, **35**, **37**, and **43** had already been reported in the genus *Gaultheria* [20,41], whereas compounds **23**, **29**, **30**, **32**, **33**, **40**–**42** and **44** were detected for the first time. In all extracts flavonoids were mainly represented by three quercetin derivatives MQ (**28**), HY (**26**) and quercetin pentoside-glucuronide (**23**). The previously published data [20] suggested that IQ (**27**) is the dominant *G. procumbens* leaf flavonoid. However, the strong elution gradient applied by Saleem *et al.* [20] resulted probably in co-elution of the critical bands of IQ and MQ. The co-elution tendency was observed also by our team, especially when the strong gradient profile was used, similar to that applied in [20]. Optimization of the more selective elution profile for UHPLC-PDA-ESI-MS*^3^*-fingerprinting resulted in high resolution between the adjacent bands (Figure 4) and enabled undoubted identification of MQ as the dominant flavonoid of the title plant.

### 2.5. Quantitative Profiling of G. procumbens Leaf Phenolics

Quantitative phenolic profile was studied by UV-spectrophotometric (total proanthocyanidins) and HPLC-PDA methods (total flavonoids and fingerprinting of individual compounds). The subsequent correlation studies were applied to select the main determinants of the tested bioactivities.

**Figure 5:**
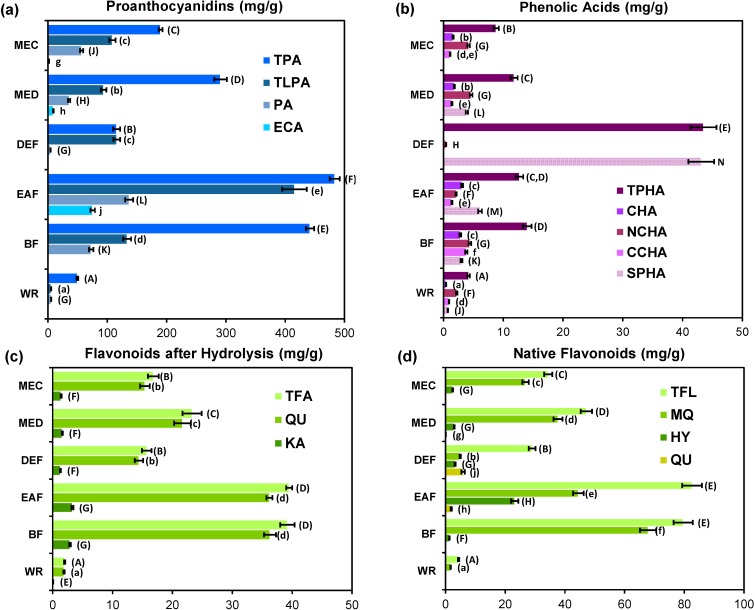
Quantitative data of *G. procumbens* dry leaf extracts: (**a**) proanthocyanidins; (**b**) phenolic acids; (**c**) flavonoid aglycones after acid hydrolysis; and (**d**) native flavonoids.

As reported in Figure 5, the highest total level of individual phenolics, as assayed by HPLC-PDA (511.0 mg/g DW), was found for EAF, which confirmed that ethyl acetate has the best ability to concentrate the bioactive constituents of *G. procumbens* leaves.

The total content of proanthocyanidins (TPA), as determined by the butanol-HCl assay and expressed in cyanidin chloride equivalents (CyE), was in the range of 49.6–483.4 mg/g DW and constituted 65.6%–100.4% of the TPC values with one outlier for DEF (21.3%), and thus prevailed strongly in the phenolic fractions of most extracts. However, this assay is not diagnostic for the degree of polymerization, and can give positive responses for molecules varying from monomers to highly condensed ones. On the other hand, RP-HPLC method enables separation of low- and medium-molecular-weight proanthocyanidins (monomers, dimers, and trimers), that are of special interest as health-promoting agents due to their beneficial profile of bioavailability and bioactivity, including antioxidant and anti-inflammatory actions [42]. All 12 individual compounds of this type identified by UHPLC-PDA-ESI-MS*^3^* (see Section 2.4), were detectable also by HPLC-PDA, including CA, ECA and the procyanidin A-type trimer (**17**), marked PA. The total content of these compounds (TLPA), calculated as ECA, varied among extracts, with the highest level found for EAF (415.7 mg/g DW). The TLPA levels constituted 10.6%–100.4% of the TPA contents, with the highest shares observed for DEF (100.4%) and EAF (86.5%). As described above (Section 2.1 and Section 2.2), DEF and EAF exhibited the highest TPC levels and the most potent activity in the majority of test systems. This close relationship was confirmed by a significant correlation between TLPA and TPC levels (*r* = 0.8675, *p* < 0.05) indicating a strong impact of TLPA on the FC-response. Similar trends were observed between TLPA levels and parameters of the DPPH (*r* = −0.9714, *p* < 0.01), inhibition of LA-peroxidation (*r* = −0.9698, *p* < 0.01), and lipoxygenase inhibition (*r* = −0.9167, *p* = 0.08) tests for most extracts except EAF, which deviated from these models by extremely high TLPA level in comparison to the exerted activity. This discrepancy could be probably connected with the flavan-3-ol profile of EAF (*i.e.*, monomers-to-oligomers ratio) and specific reactivity of individual molecules in particular tests [43].

The major A-type proanthocyanidin of *G. procumbens* (PA) was present in considerable amounts (5.3–136.9 mg/g DW) and ratios to the TLPA levels (32.9%–100%) in all extracts except DEF (Figure 5a). Excluding this extract, a strong and statistically significant (*p* < 0.05) correlations were found between PA and FRAP levels (*r* = 0.9604), H_2_O_2_ SC_50_ (*r* = −0.9013) and O_2_^•−^ SC_50_ values (*r* = −0.8216). Although weaker trends (*p* > 0.05) were observed for PA levels and DPPH EC_50_ (*r* = −0.7447), IC_50_ for LA-peroxidation (*r* = −0.7373), and LOX IC_50_ values (*r* = −0.8151), they cannot be discriminated, and PA could be indicated as an important determinant of the tested activities.

All data expressed as mean values calculated per dry weight of the extracts ± SD (*n* = 3 × 3 × 1) represented by error bars. For each parameter different letters in parentheses indicate significant differences (*p* < 0.05). Abbreviations: TPA, total proanthocyanidin content expressed in cyanidin chloride equivalents; TLPA, total content of individual proanthocyanidins expressed in epicatechin equivalents; ECA, (−)-epicatechin; PA, procyanidin A-type trimer; TPHA, total content of phenolic acids; CHA, 5-*O*-caffeoylquinic acid; NCHA, 3-*O*-caffeoylquinic acid; CCHA, 4-*O*-caffeoylquinic acid; SPHA, total content of simple hydroxybenzoic and hydroxycinnamic acids; TFA, total flavonoid content as the sum of aglycones after acid hydrolysis; TFL, total content of individual native flavonoids; QU, quercetin; KA, kaempferol; HY, hyperoside; MQ, miquelianin. All levels found by HPLC-PDA-fingerprints except for the TPA contents assayed by spectrophotometry. For extract codes see Figure 1.

The total content of native flavonoids (TFL, Figure 5d) was quite high in all extracts (29.0–82.6 mg/g DW) except WR (4.4 mg/g DW). Two flavonol aglycones (QU and KA) were found in the extracts after acid hydrolysis, with QU constituting 91.7%–94.1% of the total aglycones (Figure 5c). In the native extracts, free aglycones were minor or trace components (0.1–1.9 mg/g DW) except for QU in DEF (5.9 mg/g DW). Among flavonoids, quercetin glycosides (mainly MQ and HY) were the most abundant. Correlation studies revealed determinative impact of flavonoids on H_2_O_2_ scavenging activity due to the observed significant (*p* < 0.05) relationships between both TFL and MQ levels and H_2_O_2_ SC_50_ values (*r* = −0.8466 and *r* = −0.8426, respectively). For other antioxidant activity parameters the correlations were weaker with the lowest found for the FRAP levels (*r* = 0.6066 and *r* = 0.2570, respectively). Lack of statistical significance of these trends at α = 0.05 suggested that the impact of flavonoids on the antioxidant capacity of *Gaultheria* extracts is more additive than determinative. On the other hand, significant linear relationships between the LOX IC_50_ values and TFL levels (*r* = −0.9651, *p* < 0.01) suggested, that flavonoids are mainly responsible for enzyme mediated anti-inflammatory activity of the extracts. Although weaker than the total flavonoid effect, the impact of MQ on this activity (*r* = −0.8535, *p* = 0.07) is also of note. It is worth pointing out that MQ was proved to have strong *in vivo* antioxidant and anti-inflammatory effects [44]. The presence of MQ at high levels (26.8–37.7 mg/g DW in MEC and MED) could thus partially explain the wide biological activity of the analyzed plant.

As shown in Figure 5b, with total levels of 4.2–13.9 mg/g DW, phenolic acids are minor components of most *Gaultheria* extracts except DEF (43.5 mg/g DW; 23.1% of total phenolics detected by HPLC). The most abundant compounds among them were caffeoylquinic acid isomers (3.5–11.0 mg/g DW) constituting 52.4%–83.1% of total acids. One exception to this trend was DEF, which accumulated high quantities of simple hydroxybenzoic (29.3 mg/g DW) and hydroxycinnamic (13.8 mg/g DW) acids. Due to its unique profile, DEF was excluded from correlation studies, which then revealed strong and significant (*p* < 0.05) linear relationships between the total contents of phenolic acids and LOX IC_50_ values (*r* = −0.9821), DPPH EC_50_ values (*r* = −0.9261), as well as IC_50_ values from the LA-peroxidation test (*r* = −0.8850), and FRAP values (*r* = 0.8157). Although these relationships were less evident for H_2_O_2_ and O_2_^•−^ SC_50_ values (|*r*| ≥ 0.65, *p* > 0.05), the study indicated that, despite the lower content, phenolic acids are key determinants of the tested activities.

## 3. Experimental Section

### 3.1. General

HPLC grade reagents and standards, such as bovine testis hyaluronidase; bovine serum albumin; luminal; nitrobluetetrazolium; xanthine; xanthine oxidase; hydrogen peroxide; horseradish peroxidase; lipoxygenase from soybean; 2,2-diphenyl-1-picrylhydrazyl (DPPH); 2,2'-azobis-(2-amidinopropane) dihydrochloride (AAPH); 2,4,6-tris-(2-pyridyl)-s-triazine (TPTZ); (±)-6-hydroxy-2,2,7,8-tetramethyl-chroman-2-carboxylic acid (Trolox^®^, TX); caffeic acid (CFA); gallic acid monohydrate (GA); chlorogenic acid hemihydrate (CHA); *p*-coumaric acid (*p*CA); protocatechuic acid (PCA); (+)-catechin (CA); (−)-epicatechin (ECA); kaempferol (KA); quercetin trihydrate (QU); rutin trihydrate (RT); miquelianin (MQ, quercetin 3-*O*-*β*-d-glucuronopyranoside); astragalin (AG, kaempferol 3-*O*-*β*-d-glucopyranoside); isoquercitrin (IQ, quercetin 3-*O*-*β*-d-glucopyranoside); hyperoside (HY, quercetin 3-*O*-*β*-d-galactopyranoside); linoleic acid (LA); and ascorbic acid (AA) were purchased from Sigma-Aldrich (Seelze, Germany/St. Louis, MO, USA), as were analytical grade *p*-hydroxybenzoic acid (*p*HBA); butylated hydroxyanisole (BHA); 2,6-di-*tert*-butyl-4-methylphenol (BHT); and *tert*-butyl-hydroquinone (TBHQ). Hyaluronic acid and heparin sodium were purchased from Fluka (Steinheim, Germany) and WZF Polfa (Warsaw, Poland). An HPLC grade (min. 96.5% purity) guajiverin (GV, quercetin 3-*O*-*α*-l-arabinopyranoside) standard was isolated earlier [45]. The qualitative standards of 3-*O*-caffeoylquinic acid (NCHA) and 4-*O*-caffeoylquinic acid (CCHA) were prepared by isomerization of CHA according to the known method [35]. HPLC grade solvents (acetonitrile and methanol) used for UHPLC and HPLC analyses were obtained from POCh (Gliwice, Poland). Phosphate buffered saline (PBS) was purchased from Biomed (Lublin, Poland). All other chemicals and solvents were of analytical grade and obtained from POCh (Poland). In all analyses redistilled water was used. For nonenzymatic spectrophotometric tests samples were incubated in a constant temperature using a BD 23 incubator (Binder, Tuttlingen, Germany) and measured using a Lambda 25 spectrophotometer (Perkin-Elmer, Waltham, MA, USA), in 10 mm quartz cuvettes. Enzymatic tests were done using 96-well plates and monitored using a microplate reader, Synergy 4 (BioTek, Winooski, VT, USA).

### 3.2. Plant Material and Preparation of Dry Extracts

Leaves of *Gaultheria procumbens* L. were collected in October 2012 in the nursery-garden of Ericaceae plants, Gospodarstwo Szkolkarskie Jan Cieplucha (51°44ʹN, 19°18ʹE), Konstantynow Lodzki, Poland. The seeds for the nursery were imported from the William J. Beal Botanical Garden (Michigan State University, East Lansing, MI, USA), and authenticated in the Arboretum, Forestry Experimental Station of Warsaw University of Life Sciences (SGGW) in Rogow, Poland. The voucher specimen was deposited in the herbarium of the Department of Pharmacognosy, Medical University of Lodz, Poland with the number KFG/HB/12005-GPRO.

The raw material was air-dried under normal conditions, powdered with an electric grinder, and sieved through a 0.315-mm sieve. Two portions (100 g each) were sampled, and one of them was extracted with chloroform (1 L, 48 h) in a Soxhlet apparatus to obtain the chloroform extract (CHE, 7.4 g DW). Both samples were next separately refluxed triply with 75% (*v*/*v*) aqueous methanol (1 L, 8 h) to give the crude methanol extract (MEC, 42.5 g DW) and the defatted methanol extract (MED, 39.4 g DW), respectively. The MED (35.0 g) was suspended in water (1 L) and subjected to sequential liquid-liquid extraction with organic solvents (8 × 100 mL each) to yield diethyl ether fraction (DEF, 0.7 g DW), ethyl acetate fraction (EAF, 2.5 g DW), *n*-butanol fraction (BF, 11.9 g DW) and water residue (WR, 16.3 g DW). The organic solvent extracts were evaporated *in vacuo*, and the water containing fractions were lyophilized using an Alpha 1–2/LD Plus freeze dryer (Christ, Osterode am Harz, Germany) before weigh in. The extraction process was illustrated in Figure S1.

### 3.3. Determination of Total Phenolic Content (TPC)

The total phenolic content (TPC) was determined according to the Folin-Ciocalteu method as described earlier [46] with the use of methanol/water (70:30, *v*/*v*) solutions of the tested extracts (0.12–0.90 mg/mL). Results were expressed as gallic acid equivalents (GAE) per dry weight of the extracts.

### 3.4. Biological Activity Testing

#### 3.4.1. Reactive Oxygen Species Scavenging Tests

The DPPH scavenging activity was determined based on the earlier optimized method [45] with the use of serial dilutions of the analytes (2–160 μg/mL) in methanol/water (70:30, *v*/*v*). Finally, the concentration of the analytes in the reaction medium (in µg/mL) was plotted against the percentage of remaining DPPH using the DPPH calibration curve, and the normalized EC_50_ values were calculated as described previously [47].

The xanthine/xanthine oxidase system with nitrobluetetrazolium (NBT) reduction, was used to determine the superoxide anion (O_2_^•−^) scavenging capacity as described previously [48]. Prior to the analysis, the extracts were dissolved in a (Ca^2+^)-free PBS buffer to the concentrations of 2.5–400.0 μg/mL. As a positive control, ascorbic acid was used. Finally, the SC_50_ values were calculated from the calibration curves. In order to assess the possibility of direct interaction of the tested analytes with xanthine oxidase, the uric acid production by the enzyme was monitored at 295 nm [49].

The hydrogen peroxide (H_2_O_2_) scavenging capacity was tested by the horseradish peroxidase method as described previously [48]. Prior to the analysis, the extracts were dissolved in the (Ca^2+^)-free PBS buffer to the concentrations of 10–140 μg/mL. As a positive control, quercetin was used. Finally, the SC_50_ values were calculated from the five-point calibration curves.

#### 3.4.2. Ferric Reducing Antioxidant Power (FRAP) Assay

The FRAP was determined according to [30] with some variations [47]. Prior to the analysis, the analytes were diluted with methanol/water (70:30, *v*/*v*) to the concentrations of 40–360 μg/mL. The activity was expressed in micromoles of ferrous ions produced by 1 g of the dry extract or standard, which value was calculated from the eight-point calibration curve of ferrous sulphate.

#### 3.4.3. Linoleic Acid Peroxidation Test (Ferric Thiocyanate Method, FTC)

The ability of the analytes to inhibit AAPH-induced LA-peroxidation was assayed as described earlier [47] and with the use of serial dilutions of the analytes (0.07–3.00 mg/mL) in methanol/water (70:30, *v*/*v*). Finally, the IC_50_ values were calculated from the five-point concentration-inhibition calibration curve.

#### 3.4.4. Hyaluronidase and Lipoxygenase Inhibition Tests

Inhibition of hyaluronidase was determined by the modified turbidimetric USP [50] with heparin used as a positive control. Prior to the assay, the analytes were dissolved in monosodium phosphate buffer (pH = 7.0) with 0.01% BSA to the concentrations of 1–2 mg/mL.

Inhibition of lipoxygenase was tested according to [48] with the use of serial dilutions of the analytes (100–600 μg/mL) in sodium borate buffer (pH = 9.0). As a positive control, quercetin was used. Finally, the IC_50_ values were calculated from the five-point calibration curves.

### 3.5. Phytochemical Profiling

#### 3.5.1. UHPLC-PDA-ESI-MS*^3^* and HPLC-PDA Fingerprint Conditions

The UHPLC-PDA-ESI-MS*^3^* analysis was performed using an UHPLC-3000 RS system (Dionex, Dreieich, Germany) equipped with a dual low-pressure gradient pump, an autosampler, a column compartment, a diode array detector, and an AmaZon SL ion trap mass spectrometer with an ESI interface (Bruker Daltonik, Bremen, Germany). Separations were carried out on a Kinetex XB-C18 column (1.7 μm, 150 mm × 2.1 mm i.d.; Phenomenex, Torrance, CA, USA) at 25 °C. The mobile phase (A) was water/acetonitrile/formic acid (95:5:0.1, *v*/*v*/v), and the mobile phase (B) was acetonitrile/formic acid (100:0.1, *v*/*v*). A linear gradient system was used for elution: 0–45 min, 6%–26% B (*v*/*v*); 45–55 min, 26%–95% B; 55–63 min, 95% B; 63–70 min, 95%–6% B; 70–80 min, 6% B (equilibration). The flow rate was 0.3 mL/min. Before injection, samples of the tested extracts (10–40 mg) were dissolved in 70% (*v*/*v*) aqueous methanol (10 mL) and filtered through a PTFE syringe filter (13 mm, 0.2 µm, Whatman, Pittsburgh, PA, USA). UV-Vis spectra were recorded over a range of 200–600 nm, and chromatograms were acquired at 254 nm, 280 and 350 nm. LC eluate was introduced directly into the ESI interface without splitting and analyzed in a negative and positive ion mode. MS*^2^* and MS*^3^* fragmentations were obtained for the most abundant ions at the time. The nebulizer pressure was 40 psi; dry gas flow 9 L/min; dry temperature 300 °C; and capillary voltage 4.5 kV. Analysis was carried out using scan from *m*/*z* 200 to 2200.

The HPLC-PDA assays were carried out on a Waters 600E Multisolvent Delivery System (Waters, Milford, MA, USA) with a PDA detector (Waters 2998) detector scanning in the wavelength range of 220–450 nm; a model 7725 sample injection valve (Rheodyne, Pittsburgh, PA, USA); and a 5 μL injection loop. A C18 Ascentis^®^ Express column (2.7 μm, 75 mm × 4.6 mm i.d.; Supelco, Bellefonte, PA, USA), guarded by a C18 Ascentis^®^ C18 Supelguard guard column (3 μm, 20 mm × 4 mm i.d.; Supelco), was maintained at 30 °C using a Jetstream Plus 5480 thermostat (Thermotechnic Products, Langenzersdorf, Austria). An injection volume of the samples (prepared as described above) was 5 µL. The analytical method was the same as applied previously for *Sorbus* extracts [47] with twelve external standards used for calibration including CA, ECA, CFA, CHA, *p*CA, PCA, *p*HBA, RT, HY, IQ, MQ, QU, and KA. Identification and peak purity tests were made with an automated match system (Waters Empower 2 PDA software). The tentatively identified peaks were quantified as equivalents of ECA (proanthocyanidins), PCA (hydroxybenzoic acids), CHA (chlorogenic acid isomers), CFA or *p*CA (hydroxycinnamic acid derivatives other than CHAs, depending on their UV-Vis spectra), RT (flavonoid diglycosides, mean flavonoids eluting before RT), and IQ (flavonoid monoglycosides, mean flavonoids eluting after RT).

#### 3.5.2. Determination of Total Contents of Main Groups of Polyphenols (TPA and TFA)

The total proanthocyanidin content (TPA) was quantified by the modified butanol-HCl assay as described previously [47] with the use of methanol/water (70:30, *v*/*v*) solutions of the tested extracts and fractions (0.40–2.00 mg/mL). The results were expressed as cyanidin chloride (CYE) equivalents per dry weight of the extracts.

The total flavonoid content (TFA) was determined by HPLC-PDA as the total content of flavonoid aglycones after acid hydrolysis, according to [51] with some modifications. Samples of the extracts (50–150 mg) were heated under reflux for three hours with 70% (*v*/*v*) aqueous methanol (30 mL) and 25% (*w*/*v*) hydrochloric acid (9 mL). The hydrolysate was diluted with methanol to 50 mL, filtered through a PTFE syringe filter, and injected (5 µL) into the HPLC system (see Section 3.5.1). The elution system consisted of solvent A (0.5% water solution of orthophosphoric acid, *w*/*v*) and solvent B (methanol), with the elution profile as follows: 0–2 min, 40% B (*v*/*v*); 2–5.5 min, 40%–60% B; 5.5–6 min, 60%–40% B; 6–10 min, 40% B (equilibration). The flow rate was 1.3 mL/min, and the column was maintained at 40 °C. Aglycones were identified by PDA-spectra and detected at 370 nm. Two external standards, QU and KA, were used for calibration.

### 3.6. Statistical Analysis

Results were reported as means ± SD (standard deviation) of the indicated number of experiments. The statistics (calculation of SD, one-way analysis of variance, HSD Tukey’s tests, and linearity studies) were performed using the software Statistica10Pl for Windows (StatSoft Inc., Krakow, Poland) with *p* values less than 0.05 regarded as significant.

## 4. Conclusions

Leaves of *G. procumbens* contain considerable quantities of antioxidant polyphenols, which can be effectively extracted with 75% (*v*/*v*) aqueous methanol and further concentrated by ethyl acetate. The obtained extracts possess significant and dose-dependent SET- and HAT-type antioxidant capacities *in vitro*, as well as moderate anti-inflammatory activity, which correlate with their phenolic composition. Among the phenolic constituents, low- and medium-molecular-weight proanthocyanidins and phenolic acids are primarily responsible for the tested antioxidant capacity, whereas flavonoids are the main determinants of the anti-inflammatory effects. Regarding the potent activity of *Gaultheria* extracts in comparison with phenolic standards, the results of the present study partly explain the ethnobotanical use of the plant and support the idea that Ericaceae leaves may be good sources of beneficial prophylactic phenolic antioxidants with anti-inflammatory potential. However, further research is needed to confirm the present results in cell-based or *in vivo* studies, and to investigate in detail the dominant phenolic constituents of the analyzed extracts, especially proanthocyanidins, in terms of their structure and biological effects at molecular levels.

## Supplementary Materials

Supplementary materials can be accessed at: http://www.mdpi.com/1420-3049/19/12/20498/s1.
