# Supplementary material for: Polyphenolic Profile, Antioxidant and Anti-Inflammatory Activity of Eastern Teaberry (Gaultheria procumbens L.) Leaf Extracts
Source: Molecules. 2014 Dec 8;19(12):20498–520. doi: 10.3390/molecules191220498 (PMC6271927; doi:10.3390/molecules191220498)
Supplement: Supplementary file 1 [file molecules-19-20498-s001.pdf]

Supplementary Materials

Figure S1. Scheme illustrating the process of sequential extraction and fractionation of the dried leaves of *Gaultheria procumbens* L.

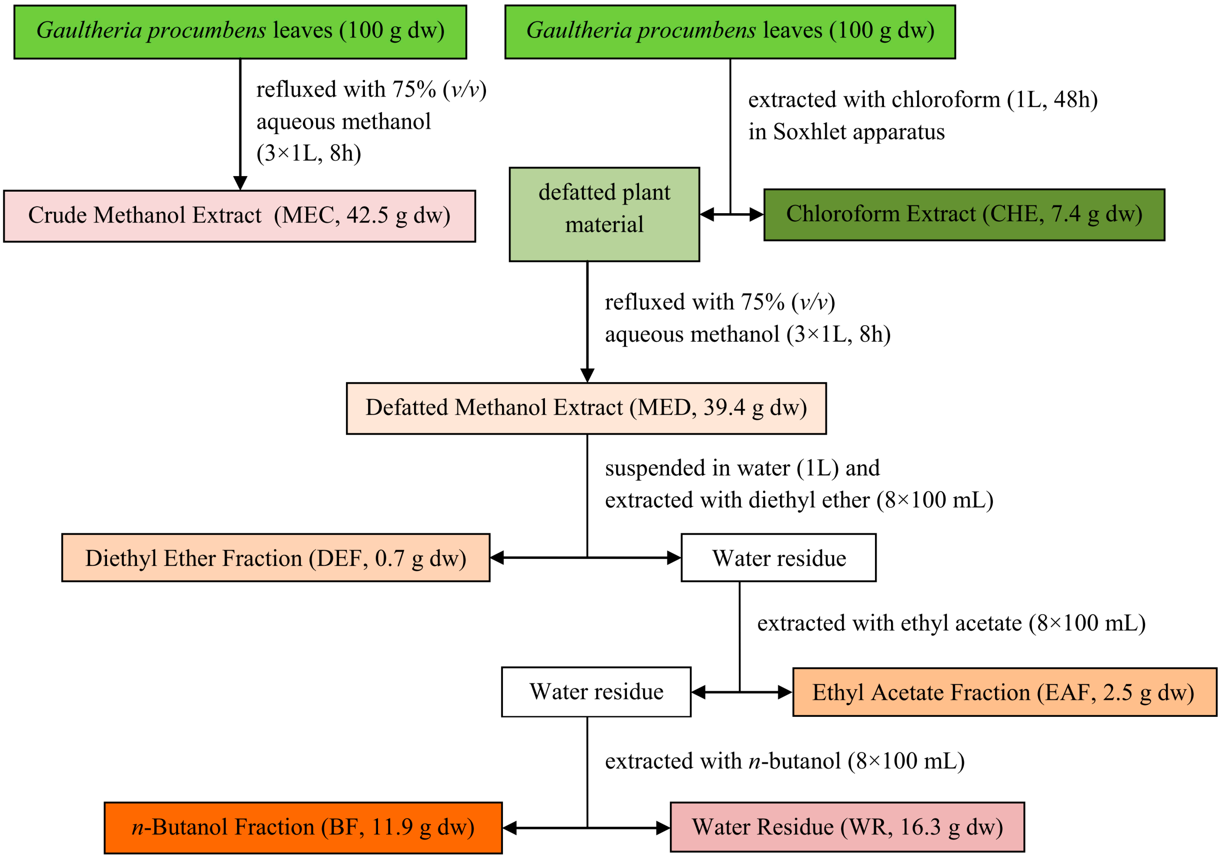

Table S1. Retention times, UV-Vis and MS^3 data for compounds detected in the analyzed *Gaultheria* extracts.

	Compounds	Retention Time (min)	UV λ_{max} (nm)	$[M–H]^-$ *m/z*	MS^2 ions	MS^3 ions	$[M+H]^+$ *m/z*	MS^2 ions	MS^3 ions
**1**	protocatechuic acid (PCA) [a]	4.4	295	153			155		
**2**	3-*O*-caffeoylquinic acid (neochlorogenic acid, NCHA) [a]	6.2	325	353	191, 179		377 [b]	359, 215	
**3**	*p*-hydroxybenzoic acid (*p*HBA) [a]	7.6	254	137	93		139		
**4**	3-*O*-*p*-coumaroylquinic acid [a]	9.3	310	337	163		339	147	
**5**	vanillic acid [a]	10.3	260, 291	167			169		
**6**	5-*O*-caffeoylquinic acid (chlorogenic acid, CHA) [a]	10.7	325	353	191, 179		377 [b]	359, 215	
**7**	(+)-catechin (CA) [a]	10.9	280	289	245, 205		291	165, 139, 123	
**8**	caffeic acid (CFA) [a]	11.7	325	179	135		181		
**9**	4-*O*-caffeoylquinic acid (cryptochlorogenic acid, CCHA) [a]	12.6	325	353	173		377 [b]	359, 215	
**10**	3-*O*-feruloylquinic acid	14.2	325	367	193		369	163	
**11**	procyanidin B-type dimer	14.9	280	577	425, 407, 289	407, 273	579	427, 409, 291	409, 301, 275
**12**	unknown compound	15.7	254	481	385, 345, 327, 165, 153		483		
**13**	(−)-epicatechin ECA) [a]	16.5	280	289	245, 205		291	165, 139, 123	
**14**	4-*O*-*p*-coumaroylquinic acid	16.6	310	337	173		339		
**15**	unknown compound	17.3	280	559	542, 445, 407, 293, 265		561	295, 259, 153	
**16**	*p*-coumaric acid (*p*CA) [a]	19.1	310	163	119		165		
**17**	procyanidin A-type trimer (PA)	19.8	280	863	711, 693, 573, 559, 451, 411, 289	693, 559, 407, 285	865	713, 695, 575, 533, 453, 411, 287	695, 543, 409, 287
**18**	procyanidin B-type trimer	20.8	280	865	713, 695, 577, 407, 287	695, 561, 407, 245	867	715, 699, 579, 427, 407, 287,	697, 545, 425, 245
**19**	procyanidin A-type dimer	22.1	280	575	499, 423, 407, 289		577	492, 407, 289, 165	
**20**	unknown compound	22.2	267, 298	639	319, 183	183, 165, 153	641		
**21**	caffeoylquinic acid derivative	22.4	325	391	179		393		

Table S1. *Cont.*

	Compounds	Retention Time (min)	UV λ_{max} (nm)	$[M–H]^−$ *m/z*	MS^2 ions	MS^3 ions	$[M+H]^+$ *m/z*	MS^2 ions	MS^3 ions
**22**	unknown compound	24.3	280	473	427, 293		475	457, 431, 249, 166	
**23**	quercetin pentoside-glucuronide	24.9	257, 356	609	482, 301	301	611	479, 303	303
**24**	procyanidin A-type trimer	25.1	280	863	711, 573, 559, 451, 411	693, 559, 542, 407	865	713, 533, 407, 287	695, 543, 409, 287
**25**	unknown compound	26.3	280	451	341		453	343, 301, 191	191
**26**	quercetin 3-*O*-galactoside (hyperoside, HY) [a]	27.2	254, 353	463	301	271, 255, 179, 151	465	303	285, 257, 229, 165
**27**	quercetin 3-*O*-glucoside (isoquercitrin, IQ) [a]	28.1	256, 353	463	301	271, 255, 179, 151	465	303	285, 257, 229, 165
**28**	quercetin 3-*O*-glucuronide (miquelianin, MQ) [a]	28.8	256, 356	477	301	273, 257, 179, 151	479	303	285, 257, 229, 165
**29**	quercetin 3-*O*-arabinoside (guajiverin, GV) [a]	30.3	258, 356	433	301	271, 255, 229, 179, 151	435	303	285, 257, 229, 165
**30**	quercetin derivative	30.6	258, 354	333	301, 165	165	335	303, 137	
**31**	kaempferol 3-*O*-glucuronide	33.0	265, 349	461	285	257, 229, 163, 151	463	287	259, 231, 213, 165, 153
**32**	quercetin 3-*O*-glucuronide methyl ester	33.9	265, 356	491	473, 315, 301	301	493	303	
**33**	kaempferol 3-*O*-glucuronide methyl ester	38.8	265, 348	475	327, 285, 255		477	287	
**34**	unknown compound	39.0	286, 326	409	335, 235, 161		411	393, 249, 163	
**35**	kaempferol 3-*O*-glucoside (astragalin, AG) [a]	39.9	265, 345	447	285		449	287	231, 213, 165
**36**	unknown compound	40.3	280	451	341, 217		453	343, 301	191
**37**	quercetin (QU) [a]	43.3	255, 364	301	273, 257, 229, 179, 159		303	285, 257, 229, 165	
**38**	unknown compound	43.7	286, 326	409	335, 235, 179, 161, 135		411	393, 249, 163	
**39**	unknown compound	44.4	280	435	341, 297		437	343, 285, 191	191
**40**	quercetin 3-*O*-pentoside-glucuronide butyl ester	46.8	268, 359	665	647, 533, 357, 301	515, 357, 301, 178	667	535, 303, 215	303, 215
**41**	quercetin 3-*O*-glucuronide butyl ester	48.8	256, 356	533	515, 357, 301, 179	301	535	303	
**42**	kaempferol 3-*O*-pentoside-glucuronide butyl ester	50.1	268, 350	649	631, 533, 499, 285, 255		651	519, 287, 215	287
**43**	kaempferol (KA) [a]	50.4	265, 364	285			287		
**44**	kaempferol 3-*O*-glucuronide butyl ester	50.6	265, 350	517	285, 255		519	287	

Notes: [a] comparisons with authentic standard have been made; [b] $[M+Na]^+$; in bold—ions subjected to MS^3 fragmentation.
